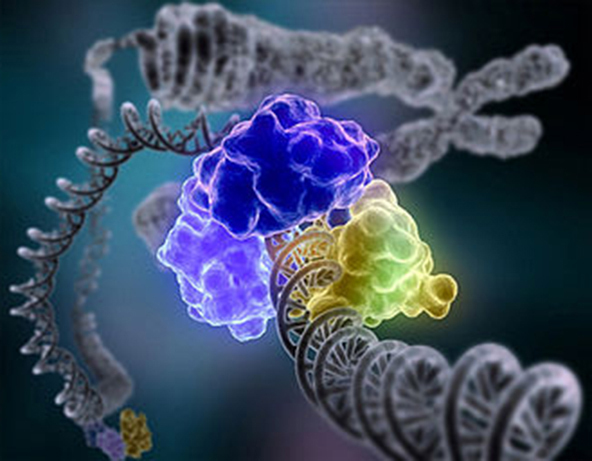# Cre-recombinase-associated toxicity highlights limitations of genome editing

**Published:** 2013-11

Bacterial endonucleases have become powerful tools for mammalian genome editing, and new technologies such as TALENs and CRISP/Cas9 are increasingly used in biomedical research. However, unwanted ‘off-target’ effects of these nucleases could limit their usefulness. The Cre-*loxP* site-specific recombination system is a long-established tool for genetic manipulation, yet recent research has suggested that this approach could also lead to adverse effects. Cre activity, which is induced in response to tamoxifen treatment, has been frequently exploited in mouse models of heart disease to investigate the effects of knocking out disease-associated genes in specific cardiac cells. Two articles published in this issue report results of investigations into Cre-recombinase-associated toxicity in mouse cardiac tissue. Nadia Rosenthal’s group systematically characterised the effects of Cre activity on cardiac morphology, physiology and function (**page 1470**). Their analysis revealed that a significant proportion of treated mice develop cardiac fibrosis in response to Cre activity. Interestingly, the severity of the phenotype varied in different genetic backgrounds, and toxicity was dependent on the frequency of tamoxifen injections. In the second study, Bernhard Kühn and colleagues substantiate these findings by demonstrating that Cre activity leads to cardiac toxicity and cardiomyocyte apoptosis in a dose-dependent manner (**page 1459**). Importantly, both studies ruled out the possibility that toxicity is caused by tamoxifen rather than Cre activity. Collectively, these analyses confirm that the Cre-*loxP* system can have unwanted, toxic effects in target tissue, highlighting the need for careful experimental design in studies utilising this tool and related genome-editing technologies.

**Figure:**